# Beyond ‘seeing is believing’: the antenna size of the photosystems *in vivo*

**DOI:** 10.1111/nph.16758

**Published:** 2020-07-14

**Authors:** Roberta Croce

**Affiliations:** ^1^ Biophysics of Photosynthesis Department of Physics and Astronomy Faculty of Science Vrije Universiteit Amsterdam De Boelelaan 1083 Amsterdam 1081 HV the Netherlands

**Keywords:** light‐harvesting complexes, photosynthesis, Photosystem I, Photosystem II, thylakoid membrane

## Abstract

Photosystems I and II are the central components of the solar energy conversion machinery in oxygenic photosynthesis. They are large functional units embedded in the photosynthetic membranes, where they harvest light and use its energy to drive electrons from water to NADPH. Their composition and organization change in response to different environmental conditions, making these complexes dynamic units. Some of the interactions between subunits survive purification, resulting in the well‐defined structures that were recently resolved by cryo‐electron microscopy. Other interactions instead are weak, preventing the possibility of isolating and thus studying these complexes *in vitro*. This review focuses on these supercomplexes of vascular plants, which at the moment cannot be ‘seen’ but that represent functional units *in vivo*.

**Table:** 

	Contents
	Summary	1
I.	Introduction	1
II.	The antenna size of Photosystem II	2
III.	The antenna size of Photosystem I	2
IV.	PSI, PSII and PSI–PSII megacomplexes	4
V.	Concluding remarks	4
	Acknowledgements	4
	References	4

## I. Introduction

Photosystem I (PSI) and Photosystem II (PSII) are large assemblies of many pigment–protein complexes and can be divided into two functional parts: the core and the antenna. The cores are highly conserved in all organisms performing oxygenic photosynthesis and contain the reaction center (RC), where photochemistry occurs. The role of the antenna is to collect light and transfer the excitation energy to the RC. The antenna varies in composition and organization between organisms, and in plants it is composed of members of the light‐harvesting complex (LHC) multigenic family (Pan *et al*., 2020).

The large number of high‐resolution observations of photosystem structures published in recent years has provided new information regarding their supramolecular organization and the interactions between complexes in plants (Su *et al*., 2017; Pan *et al*., 2018) as well as in several algae (e.g. Pi *et al*., 2019; Sheng *et al*., 2019; Suga *et al*., 2019). While there is no doubt that the purified complexes exist in the membrane, it is also true that the thylakoid membrane is a dynamic system that is able to change in response to different environmental conditions (Chow *et al*., 1990; Johnson & Wientjes, 2019). Moreover, the protein density in the thylakoid membrane can be as large as 70% (Haferkamp *et al*., 2010), meaning that each complex is surrounded by other complexes with which it might or might not functionally interact. Some of these interactions are controlled via phosphorylation, which is induced by different environmental factors (Goldschmidt‐Clermont & Bassi, 2015; Rantala *et al*., 2020). This makes the ‘photosystems’ dynamic entities which can change their composition and organization depending on the conditions. While some of the interactions between the complexes are strong enough to survive purification, and can thus be observed in the high‐resolution structures, others are weak and are lost during purification. The result of these effects is that not all functional units could (thus far) be isolated and analyzed *in vitro*. The large photosynthetic complexes in particular can, at the moment, only be studied *in vivo* or *ex‐vivo*. Cryo‐electron tomography (Daum *et al*., 2010; Wietrzynski *et al*., 2020) and atomic force microscopy (AFM) (Phuthong *et al*., 2015; Wood *et al*., 2018) were shown to be able to map the organization of the complexes in the membrane thanks to the fact that several core subunits protrude out of the membrane. This is not the case for the light‐harvesting complexes, which have flat surfaces and cannot be directly visualized, although recent results on LHCII membranes show the potential of AFM to detect the small LHCII protrusions (Adams *et al*., 2018). However, at the moment, it is often assumed that the LHCs occupy most of the space between the cores.

If we cannot see the LHCs, how do we know that they are there? Biochemical and functional data show that the ratio of LHC : core complexes and the number of pigments per RC are higher than what is reported for the purified (super)complexes, suggesting that the antenna size of the photosystems is larger *in vivo* (e.g. Melis & Anderson, 1983; Kouril *et al*., 2013). Time‐resolved fluorescence measurements show that the excited‐state lifetimes of the photosystems *in vivo* are longer than those of the purified complexes, indicating that indeed they contain a larger number of pigments (e.g. Wientjes *et al*., 2013b; Chukhutsina *et al*., 2020). This is due to the fact that the time between the absorption of a photon by one antenna Chl and its energy utilization in the RC for charge separation (trapping time) scales roughly with the number of Chls in the antenna (see Van Amerongen *et al*., 2000, Chapter 1, for details).

## II. The antenna size of Photosystem II

**Fig. 1:**
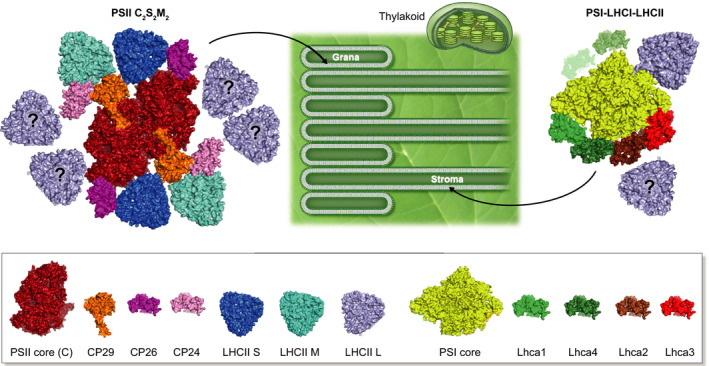
Photosystem I (PSI) and Photosystem II (PSII) structures and their locations in the membrane. The structure of PSII C_2_S_2_M_2_ (Su *et al*., 2017) is shown on the left hand side of the image; a schematic representation of the thylakoid membrane with (stacked) grana and stroma lamellae is shown in the centre; and the structure of PSI‐LHCI‐LHCII (Pan *et al*., 2018) is shown on the right. The LHCII trimers indicated with a question mark (‘?’) are not observed in the structures but are present in the membranes, but their exact position is unknown. The Lhca1 and Lhca4 observed by Crepin *et al*. (2020) in some of the PSI particles are represented in lighter shades of green. The arrows indicate the locations of PSII and PSI in the grana and stroma lamellae, respectively.

The outer antenna of plant PSII is composed of several trimeric LHCII complexes, as well as three monomeric LHCs, namely CP29, CP24 and CP26, which in most cases are present in a 1 : 1 stoichiometry with the core (Fig. 1). The number of LHCII trimers instead depends on the organism and on the light conditions. Plants grown in low light increase the number of LHCIIs to maximize light absorption, while this number decreases in high light to minimize photodamage (Anderson & Andersson, 1988). Arabidopsis plants modulate their antenna size by changing the expression of two main proteins: Lhcb1 and Lhcb2, which are the main components of the LHCII trimers (Ballottari *et al*., 2007). The number of LHCII trimers was calculated to go up to four per RC in low light in Arabidopsis (Kouril *et al*., 2013). This number is very similar to what can be extracted using the pigment/RC data from functional measurements (235 Chls per RC (Melis & Anderson, 1983)) considering the now‐known pigment‐binding stoichiometry of the complexes. The available high‐resolution structures of plant PSII supercomplexes contain one (C_2_S_2_ supercomplex; Wei *et al*., 2016) or two (C_2_S_2_M_2_; Su *et al*., 2017) LHCII trimers per monomeric core (C). While trimer S (strongly bound) seems to be well connected to the core, trimer M (moderately bound) is less stably associated with the complex, and indeed, its position is less well defined in the structure (Su *et al*., 2017). Dissociation of this trimer was observed during high light exposure, and it was suggested to have a role in photoprotection (Betterle *et al*., 2009). A low‐resolution PSII supercomplex with an extra LHCII trimer (named L, loosely bound) was observed upon solubilization of the membrane (Boekema *et al*., 1999). More recently, L‐LHCII was observed in PSII megacomplexes, where its binding is probably stabilized by the presence of two adjacent PSII complexes (Nosek *et al*., 2017). A complex with three LHCII per core was obtained from the green alga *Chlamydomonas reinhardtii(e.g*.(Sheng *et al*., 2019)), where the association of the third trimer is stronger, probably because of the absence of CP24 in this organism. However, *C. reinhardtii* is known to have an even larger number of LHCII per core *in vivo* (Drop *et al*., 2014).

The functional connection of the extra trimers to the core was confirmed by time‐resolved spectroscopy on the membranes. While the trapping time of the isolated C_2_S_2_M_2_ supercomplex was 158 ps (Caffarri *et al*., 2011), this value increased to 180, 225 and 310 ps in membranes with 2.2, 2.4 and 3.1 LHCII (Wientjes *et al*., 2013b) and to 340 ps the membranes with 4 LHCII per RC (van Oort *et al*., 2010). These results indicate that the additional LHCII trimers are a functional antenna of PSII, being well‐connected to the core to which they transfer excitation energy with high efficiency.

## III. The antenna size of Photosystem I

The ‘classical’ plant PSI complex is composed of a core and 4 LHCI (Lhca1–4) in a 1 : 1 stoichiometry. This complex is very stable and its structure was resolved in 2003 by X‐ray crystallography (Ben‐Shem *et al*., 2003). There are two more *Lhca* genes in the genome of Arabidopsis, *Lhca5* and *Lhca6*, but they are expressed at a low level. They were found in the PSI‐NDH complex, and it has been suggested that in this complex Lhca5 substitutes for Lhca4, and Lhca6 for Lhca2 (Otani *et al*., 2018). This is in agreement with the observation that in the Lhca4 knockout mutant, the position of Lhca4 can be occupied by Lhca5. All other Lhcas are not able to substitute for each other, and the absence of one of the subunits was shown to leave a ‘hole’ in the structure, indicating that the docking sites are specific for each complex (Wientjes *et al*., 2009). However, recently a PSI particle containing one additional Lhca1–4 dimer was reported (Crepin *et al*., 2020). This dimer is located on the side of the core, opposite to LHCII, a position that in *C. reinhardtii* is partially occupied by Lhca2 and Lhca9 (Suga *et al*., 2019) and that was supposed to be accessible only in green algae. This finding has several implications regarding the plasticity of plant PSI: first, the stoichiometry Lhca : core is not fixed; second, the same Lhcas can dock to the core in different positions; and third, there are more than four binding sites for Lhca around the core.

In addition to the Lhcas, the antenna of plant PSI can be enlarged by the association of one LHCII trimer to the core to form what is called PSI‐LHCI‐LHCII (Pan *et al*., 2018) (Fig. 1). This complex is related to state transitions, a process that rebalances the excitation of the two photosystems in response to changes in the color of the available light. In light conditions favoring PSII, the STN7 kinase is activated and phosphorylates LHCII, which then functionally disconnects from PSII and connects to PSI in what is called state‐1‐to‐state‐2 transition. The reverse transition is due to the action of the PPH1 phosphatase: dephosphorylated LHCII disconnects from PSI and associates with PSII (see Goldschmidt‐Clermont & Bassi, 2015 for details). The mobile LHCII is a loosely bound L trimer. Interestingly, the association of this trimer with PSI survives purification (Galka *et al*., 2012), while, as mentioned in the previous paragraph, this is not the case for its association with PSII. The transfer of excitation energy from this trimer to PSI is also faster than to PSII (compare Wientjes *et al*., 2013a; Wientjes *et al*., 2013b). These data indicate that the association of the mobile trimer is both structurally and functionally stronger with PSI than with PSII.

State transitions are a short‐term response to changes in light color, while the long‐term response to the same effects is an adjustment of the PSI : PSII ratio (Chow *et al*., 1990). This, together with the fact that LHCII is historically considered to be an antenna of PSII (it is encoded by *Lhcb* genes, which are defined as PSII‐related genes), might give the impression that LHCII moves to PSI only in particular conditions, while usually it is associated with PSII, to which it ‘moves back’ during the state‐2‐to‐state‐1 transition. However, it was shown that part of the mobile LHCII pool is associated with PSI in most light conditions (Wientjes *et al*., 2013a). One exception is sudden high light, in which no PSI‐LHCI‐LHCII complex was detected. However, after a few hours of high‐light acclimation, this complex is already present again in the membranes (Wientjes *et al*., 2013a). It was recently shown that in the dark there is also an LHCII population that is functionally connected to PSI (Chukhutsina *et al*., 2020). Finally, even in far‐red light, which is known to be mainly absorbed by PSI, part of the mobile LHCII population is associated with PSI (Bos *et al*., 2019). It can thus be concluded that LHCII is a constitutive antenna complex of Photosystem I and that PSI‐LHCI‐LHCII is probably the most representative PSI complex *in vivo*. Knowing that PSI‐LHCI contains 158 Chls and one trimer of LHCII 42 (Pan *et al*., 2018), this is in agreement with the data of Melis & Anderson (1983), which showed that the antenna of PSI in spinach is composed of 210 Chls.

Recent results show that more than one LHCII trimer can be associated with PSI. Patches of membranes with up to five LHCII trimers were purified from spinach using styrene–maleic acid copolymer (Bell *et al*., 2015), and it was shown that at least three of these LHCII trimers are active in transferring energy to PSI (Bos *et al*., 2017). Particles in which more than one LHCII trimer was associated with PSI were also observed by electron microscopy, although in a very low amount (Yadav *et al*., 2017). Are these complexes a good representation of PSI *in vivo*? Benson et al. concluded that in addition to the LHCII associated with PSI on the K side of the core, LHCII trimers in state 2 dock to PSI via the Lhca (Benson *et al*., 2015). This is in agreement with recent results, which demonstrate that in state 2 more than one LHCII trimer is associated with PSI in the model plant Arabidopsis (Bos *et al*., 2019; Chukhutsina *et al*., 2020). Time‐resolved measurements of several plant species showed that the PSI excited‐state lifetime *in vivo* varies between 72 ps in tobacco and 147 ps in maize, but it is always longer than that of the PSI‐LHCI complexes purified from the same plant (Chukhutsina *et al*., 2020). Longer excited‐state lifetimes *in vivo* were also observed for spinach (Farooq *et al*., 2018). It was concluded that depending on the species and the growth conditions, between 1 and 3 LHCII trimers can be functionally associated with PSI *in vivo*.

## IV. PSI, PSII and PSI–PSII megacomplexes

The crowdedness of the membrane also favors the interactions between photosystems (Haferkamp *et al*., 2010), which is at the basis of the PSII excitonic connectivity (see Stirbet, 2013 for a comprehensive discussion on this topic). Megacomplexes composed of either several PSI or several PSII units were observed by blue native gel and electron microscopy (Suorsa *et al*., 2015; Nosek *et al*., 2017). PSII complexes were also found in crystalline arrays in membrane preparations (Kouril *et al*., 2013). However, their function is at the moment unclear.

Do PSI and PSII interact *in vivo*? From the seminal work of Jan Anderson, it is well established that PSI and PSII are located in two distinct regions of the thylakoid membranes, the stroma lamellae and the grana, respectively (Andersson & Anderson, 1980). The spatial separation avoids energy transfer (spillover) from PSII to PSI, which would lead to the loss of part of the harvested energy (van der Weij‐de Wit *et al*., 2007). However, more recently, based on native gel analyses, it was proposed that PSI and PSII form megacomplexes at the grana margins (Suorsa *et al*., 2015; Yokono *et al*., 2015), and it was suggested that this organization facilitates the regulation of the antenna size of the two photosystems in response to phosphorylation (Grieco *et al*., 2015). Although this is an attractive proposal, it is currently difficult to conclude whether these megacomplexes exist *in vivo* and represent functional units. Electron tomography data on cells of the alga *C. reinhardtii* revealed a random organization of the photosystems and a clear separation between appressed membranes, hosting PSII, and nonappressed membranes, hosting PSI (Wietrzynski *et al*., 2020). The same technique applied to the plant cell will shine light on the organization of the complexes *in vivo* in physiologically relevant conditions.

## V. Concluding remarks

Photosystems I and II, when present *in vivo* in the thylakoid membrane in their functional state are larger than the purified forms *in vitro*. Whereas this was expected for PSII, it was somewhat surprising for PSI, the composition of which has been considered well defined for a long time. Now that this has been established, it would be very interesting to ‘see’ the photosystems *in vivo* and to relate their change in function in different environmental conditions to changes in their composition and organization. It is expected that, thanks to recent advances, a detailed description of the membrane can be obtained by integrating cryo‐electron tomography data with the high‐resolution structures of the photosystems. The structural information combined with spectroscopic data and modeling should then provide a comprehensive picture of the thylakoid membrane in action.
